# Valedictory Address

**Published:** 1848-04

**Authors:** Eleazar Parmly

**Affiliations:** New York


### ARTICLE VI.

### VALEDICTORY ADDRESS.

#### CORRESPONDENCE.

*Baltimore College of Dental Surgery,*
March 6th, 1848.

At a meeting of the graduates, at the recent commencement, held this day in the college buildings: On motion, it was

*Unanimously Resolved,* That we listened with the most profound satisfaction to the Address of Dr. Eleazar Parmly, delivered on the occasion of our commencement; and believing that its publication would do much to benefit dental science,

*Resolved,* That Drs. H. Colburn, E. W. Mason and D. G. Varney, be appointed a committee to communicate the above resolution to Dr. Parmly, with the request that he will furnish them with a copy of the Address for publication.

*Baltimore,* March 6th, 1848.

To Dr. Eleazar Parmly.

*Dear Sir,*—We take much pleasure in sending you a copy of the above resolutions, and sincerely hope that you will comply with the unanimous wish of the meeting. Very respectfully, and truly, yours,

H. COLBURN,

E. W. MASON,

D. J. VARNEY, } *Committee.*

*New York,* March 13th, 1848,

No. 1 Bond street.

Gentlemen:

I have the pleasure to acknowledge the receipt of the resolutions passed at a meeting of the graduates, at the late commencement of the Baltimore College of Dental Surgery, together with your kind note, requesting a copy of my Valedictory Address for publication. While it gives me great pleasure to comply with your request, I feel a much higher pleasure in communicating to you the great satisfaction expressed by that highly distinguished professional gentleman, Dr. E. B. Gardette of Philadelphia, chairman of the awarding committee on that occasion, and also by his eminent associates, Drs. Maynard, Hullihen and Townsend, all of whom were highly gratified at the success and prospects of the Institution, and bestowed much praise on the graduates for the efficiency and cleverness manifested in the specimens of mechanical and surgical dentistry then brought before them.

I beg you will convey to your associates, and accept for yourselves, my warmest wishes for your professional success, and for your future welfare and happiness. I am, very truly, your friend,

E. PARMLY.

To Messrs. H. Colburn, E. W. Mason, D. G. Varney, *Committee.*

### ADDRESS,

*Delivered at the Baltimore College of Dental Surgery, before the Faculty, Graduates, Students and Assembly, on the occasion of its Eighth Annual Commencement, March* 2, 1848.
By ELEAZAR PARMLY, M. D., of New York.

LADIES AND GENTLEMEN:

THE office I hold in this institution, through the courtesy of its honorable faculty, imposes on me the pleasing duty of addressing to the graduating class, such valedictory remarks, as the occasion may naturally inspire in the breast of an individual, nearly the whole of the active part of whose life, has been devoted to the practice of dental surgery.

Honored and encouraged as we are on this occasion, by the personal presence of ladies and gentlemen of this city, whose countenance and approbation would not be given to any but a good work, I would fain recall for my further encouragement, the memory of departed men of honorable renown, whose never dying spirits may be supposed to regard with interest and pleasure, the ceremonies of this occasion.

Gardette, Hudson, Hayden and Randall, where are they if not among the good and great of by-gone years, rejoicing in the rewards of lives well spent in the service of humanity. These noble minds unassisted by educational advantages such as we enjoy in the institution here established, and in which the graduating class before me have been carefully instructed, rose to unrivalled excellence in their profession, by the force of that industry, integrity and genius, which surmount all obstacles, defy all dangers, and remove all impediments in the road to fame and fortune. Encouraged by the ever enduring presence of such examples, which neither time nor death can easily efface, we come with confidence, we had almost said with pride, to speak of the institution which calls us thus together, and of the useful science for the rapid advancement of which, it has labored with success.

It may not be here considered out of place or inappropriate to call particularly to mind one whose highest pride and ambition was centered in the improvement and elevation of professional character—the design and object of this institution. *Horace Hayden*, the benevolent and truly amiable man, whose voice would have been heard here this evening, and who would have participated in these exercises, had he not been removed by the hand of death. In my last conversation with him, his whole mind was taken up in speaking of the best means of giving character, stability and encouragement to the practitioners of our art, and the young were especially the object of his friendly regard, of his high and generous consideration. And it was for their benefit, knowing that on them must depend, the usefulness, character and dignity of the profession, that he labored both in this institution, and in the American Society of Dental Surgeons, and no one can be a member of that body or a graduate of this school, without feeling that the benefit derived from both, is owing in a good degree, to the noble, generous, and self-sacrificing spirit of their late benefactor and friend, a man of genius, a lover of science, a friend to virtue, and an ornament to the profession, which this institution is designed to improve, elevate and perfect. Those who knew and loved him, will always cherish the memory of his virtues, and look forward to the time of reunion, when tears and sorrows are no more known for ever.

A few years ago the name of dental college was utterly unknown in the vocabularies of men, there had been colleges for other objects from remote antiquity, colleges for cardinals, colleges for priests, colleges academical and colleges medical, but it was reserved for the legislature of Maryland and its monumental city, to justify their enlightened regard for the public welfare, by founding a college of dental surgery, the first that has been known in the existing annals of our race.

The benefits of this enlightened and liberal policy, have been already felt for several years in many of the states of this confederacy. Young men have been sent from this institution into the field of labor understanding their work, not a single instance of empirical practice, we are bold to affirm, has ever been chargeable upon any graduate of this institution. The reason is plain, a course of studies here effects two important objects wholly incompatible with professional quackery.

In the first place it imparts so much knowledge of the true principles of the art, that the causes of empiricism are wholly removed; we are willing to believe that ignorance, which is said to be the mother of superstition, is also the fruitful parent of dental imposture. This is certainly the most charitable conclusion in which we can rest.

In the second place, a regular course of instruction in this college impresses the minds of the graduates so deeply with sentiments of scorn and contempt for all mal-practice, that great indeed must be the temptation which would ensnare them in its guilt.

So well convinced of these facts are the members of the profession generally, that the national society of dental surgeons, has a standing ordinance, which admits to its honors, without examination, any individual who has passed with credit, the trying ordeal of the Baltimore College of Dental Surgery. This is an honor conferred on no other course of dental instruction, either public or private, in Europe or America.

The graduates of this college become convinced in the course of their professional education, of the respective advantages and disadvantages of honorable and dishonorable practice of science on the one hand, of ignorance on the other. They read the works, or otherwise learn the characters of the master spirits of the profession, who would have been distinguished in any calling of human life suited to their tastes and inclination. They thus become familiar with those intellectual and moral traits of character, those habits and manners which every where secure and command success. They know why in the present day it is, that such men as Cartwright, Bell, Nasmyth, Delabarre, Brewster and Koecker in Europe, Gardette of Philadelphia, Harris of Baltimore, Maynard of Washington city, Flagg of Boston, Elliott of Montreal, Westcott of Syracuse, Foster of New York, Hullihen of Virginia, Cleveland of Charleston, Taylor of Cincinnati, and Hale of St. Louis, with many more, perhaps, of equal skill and honor have attained professional success, which has ranked them, and still ranks them, amongst the most prosperous, the most useful, and the most happy of mankind.

From the same source, connected with their professional studies, the graduates of the college learn the true cause of that disgrace and that discomfiture which justly attend upon professional imposture in all its forms. They know why it is that the Crawcours, the Mallans, and all their imitators and rivals of less talent and less enterprise but of equal empiricism, attain neither abiding wealth nor honorable fame, by the arts of imposture and knavery. The gullibility of the community, always fluctuates from subject to subject, so that like the celebrated moon-hoax, quackery of any particular kind is soon detected, and when once exposed, can dupe only those who are willing to be deceived.

This state of things reveals to us the only hope of empiricism, a meagre hope indeed, namely, that as "all fools are not yet dead," quackery itself may survive for a while both detection and disgrace.

Students educated in the Baltimore College of Dental Surgery, can never go forth into the world ignorant of the fact that the two classes of men just named, have attained their respective objects by very different means, they learn that the honorable and successful practitioner of the dental art, he who acquires fame, friends, admirers, and a fortune, as the reward of his exertions, must be in the outset and in principle, a man of inflexible integrity; quibbling, overreaching, prevaricating, deceiving are no parts of his character. His veracity, like the virtue of the wife of the Roman king must not only be irreproachable but unsuspected. He will then merit the full confidence of the public, and that which he really merits he will ultimately receive. Truth and honesty are "cities set on a hill that cannot be hid," but however truthful a man's moral character may be, if this truthfulness does not enter into his professional acts, and render them good and useful, his character is true but in part developed; he *wills* right but *does* wrong—

such a man is deficient in professional education, and he sets himself instantly to the study of his art. He finds a defect in his character which he knows how to remedy, and his honest heart prompts him to the work. No matter what obstacles short of impossibilities, stand in his path, the inner promptings of integrity impel him to surmount them, for the purpose of bringing his work into harmony with itself. If oceans intervene he braves their dangers, if labor be required he works with assiduity, if sacrifices be demanded he makes them promptly, for he separates not his love of virtue, nor his hopes of heaven from a faithful discharge of his duty to his fellow men. If his character be exalted by religious conscientiousness he is so much the more anxious to exalt the character of his works and duties, for he has heard the interrogatory from high authority: "If a man love not his brother whom he hath seen, how can he love God whom he hath not seen?"

After such a man has become throughly versed in the various duties of his calling, another valuable trait of character becomes indispensable, this is industry, concentration of energy, and effort to effect his purpose. He has become convinced that usefulness in a man's particular calling, is the best test of his sound, moral and religious character. Indefatigable industry in his calling, therefore, is the best expression of his sound, moral and religious life. With this conviction, therefore, and with affections in accordance with it, he will be industrious. If his professional labors do not absorb all his time, he will devote his leisure hours to study.

Dentistry, in its very nature, is one of the learned professions, it has its science and its literature, men of sterling genius both in this country and in Europe, have written largely and luminously upon all branches of our art, so that no student or practitioner is now under the necessity of being destitute of a dental library. Of this we have great occasion to be proud; did time permit, I would gladly give you a catalogue of such standing authors, as every dental student should carefully peruse. For it must be confessed that there would be much danger in imbibing error, if every author who has written on the subject, should be accredited. But I may safely rely on the sources of your professional education, as every way calculated to protect you from danger in this behalf.

The standing authors to whom I have just alluded, can be neglected and disregarded only by those who would degrade their profession and level its attainments to those of handicraft workmen, or mere artizans in mechanical employment. That there are already practitioners enough of this sort, the history of the few past years abundantly confirms. But, that same history will show also, that many of our professional brethren, have hailed with gratitude, and improve with eagerness, the literary and scientific labors of their talented compeers. But neither class of these honorable examples can be imitated without persevering industry. The great wonders of our world, that have been wrought by men, have been done by a proper use of time, it is this that raised the pyramids, cultivated and subdued the earth, excavated canals, built almost interminable lines of rail roads, civilized the savage, caught and chained the untamed and swift winged lightning, gave to it our entire language, and made it the faithful and meteor-like messenger of our thoughts, our feelings and our affections—for which and by which the names of our beloved Franklin and Morse will be written not only in the remote ends of the earth, but even unto the utmost end of time. Thus, the proper use of time is invariably one of the elements of greatness. It has introduced the invention of pretty much all that goes to make up our personal and present comforts. It taught us in early life to love the twilight of morning, and to take pleasure in beholding the crimson tints of the glorious sun as they dispel the shadows of the rising day.

The two qualities just named, integrity and industry, may be regarded as the two pillars standing in the portico of the temple of Solomon, which he denominated Jachin and Boaz—Beauty and Strength, or those which Samson overthrew in the vigor of his hairy strength in Philistia's temple, causing the destruction of her princes, as long as these two pillars of a man's character remain firm and erect he cannot fall, to rise perpetually must be his destiny. His honesty of design is seconded by faithfulness of execution, and with such a man ulterior triumph is made by a law of nature to be inevitable, as easily might a deep valley overtop its circumjacent hills, as such an individual come short of the smiles of fortune. It is of such an one, that a well known poet has well said,

> "On just desert let all success depend,
> And patient merit never want a friend."*

There are, indeed, other pillars in other parts of the temple of indispensable, if not of equal importance, they are essential to its usefulness, its symmetry or its beauty. Temperance, patience, gentleness of manners, purity of mind, neatness of bodily habit are a few of their familiar names.

If the structure of professional character be thus sustained, the parts subordinate will be in proportion, and the whole will be distinguished for its symmetry and beauty.

After what has been said, it scarcely needs to be remarked, that a character opposite to this one, which I have just given, namely, one in which neither honesty nor industy, nor temperance, nor patience, nor gentleness, nor purity is found, or in which several of these graces are wanting, is sure to debase and disgrace our calling, for by the acts of such a one, every high minded man is made to feel the shame that attaches to one bearing credentials of fellowship or the insignia of fraternity. Hence it is that the same poet I have already quoted thus cautions the community,

> "Beware of those whom science never taught
> The hard but useful drudgery of thought,
> For while in indolence their years have run,
> They ask the wealth that industry has won;
> Can charity for such desire success?
> No!—let them eat the bread of idleness."

The preceding remarks address themselves with equal force and pertinency to all the students of this useful institution, as well as to those of the graduating class, who have now completed their primary education; to them, in particular, I have a few words, at the close of this address, to say, which I trust will

* Brown's Dentologia be received in kindness, as the result of the experience of one who has labored long in the field which they are about to enter, and who has advanced well nigh to the end of the journey which they are about to commence.

I should do injustice to my own feelings, and also to the enterprising founders of the Baltimore College of Dental Surgery, if I were to conclude my remarks on this occasion, without expressing to them my cordial congratulation at the gratifying success of their noble undertaking. If any human enterprise deserve success, it is, undoubtedly, that which originates in a desire of extensive usefulness, and is called upon to encounter many discouragements in its progress and accomplishment. The first step in the undertaking, which resulted in the establishment of this college, was embarrassed by difficulties which would have wholly dispirited ordinary minds, and we cannot here withhold the remark, that it is exceedingly well for the world that some minds are so buoyant with hope, so elate with confidence, so pregnant with resolution and perseverance, as that no obstacle can oppose, no danger intimidate them.

It is recorded to the honor of a brave officer, that being asked by his superior whether he could take one of the enemies' batteries, he promptly replied, "I will try, sir." But I remember well, that the founders of this college began their work with still higher pledges. To those who inquired of them whether they could procure legislative authority for the foundation of their college, they confidently replied, "*We will do it, gentlemen,*" and we see how they redeemed their pledge. They procured their charter with all the privileges they desired, and we now see its happy influence in our country.

They obtained, among other privileges, the power of conferring the degree of Doctor of Dental Surgery on all the worthy graduates of this institution, and, also, on other individuals as its faculty might deem to be worthy of that honor. From this college, then, have thus issued the first dental diplomas authorised by state legislation since the authentic history of our race.

Let the honor of doing this be esteemed by themselves as a sufficient reward for the labor of its founders. But as if this were not enough, they have had the additional satisfaction of observing the beneficial effects of their labors upon the character of the profession throughout the country. The influence of this college has been very great in checking the influx of unqualified individuals into the profession, for the last several years, in addition to this it has been a radiating focus, from which true science has perpetually issued, like light and heat from the sun.

That it may long continue in these important labors is the fervent desire of all honorable members of the profession throughout the world.

In conclusion, I deem it a pleasure to express my high admiration of the zeal, faithfulness and ability of the acting professors and teachers of this seminary. The examinations which have just closed, demonstrate, conclusively, the merit and competency of those who direct the studies of this institution; and may length of days, extensive usefulness, many social enjoyments, and much domestic bliss, be the reward of their exertions. But I must not close my remarks, without grateful allusion to those intelligent fellow-citizens, who always attest their wisdom by resorting to the best skill in the dental art. There are some, nay many, in our country, who would travel hundreds of miles for the purpose of obtaining good dental advice, and operations, rather than subject themselves for a moment to the arts of empiricism. To such we tender the thanks of all the honest and honorable members of the profession.

And on this occasion we are called upon to acknowledge the repeated and gratifying courtesy of many highly respected citizens of Baltimore, in giving countenance and encouragement to this institution by their personal attendance on occasions like the present. I will only add, after thanking you all for your patient attention to the foregoing observations, hastily, and, perhaps, incoherently thrown together, that it is my ardent wish that every blessing of heaven may perpetually attend the members of the graduating class, the gentlemen undergraduates, the professors and members of the faculty and the esteemed individuals of this assembly.

GENTLEMEN GRADUATES: This may be considered not only the most interesting, but the most eventful period of your professional life. You are now to enter upon the practice and duties of an interesting profession, which rests its claims to public favor, on its general utility as a branch of the healing art. You will be esteemed, honored, respected and rewarded, as really and as fully as the members of any other department of the medical profession. But when you leave your professors, the museum, the library, the lecture room, and the laboratory of this institution, do not, I pray you, consider your education completed. Hitherto, your practice, both mechanical and surgical, has been mostly under the eye of your professors and demonstrators in both branches of dental surgery, you are now about to commence under your own guidance, and will immediately be thrown upon your own resources, to carry out in practice the principles in which you have been carefully instructed; you will have but little to encounter compared with those who commence without knowledge, and your patients will even receive, at the outset, that benefit which has hitherto been sought and only looked for, from the old and experienced practitioner. Although you look to the products of the field in which you labor for support and for the reward of your toil, you must never forget that the field is one of humanity, requiring the utmost care and gentleness in alleviating pain and suffering, and the constant exercise of patience and forbearance with your willing but timid patrons; you will thus, by a deportment uniformly decorous and gentlemanly, meet with marked good will, and secure from all respect and kindly feeling. The time has gone by, in this country at least, (thanks be to the high-minded men that have gone before us,) when other professions, the merchant princes, and other magnates of the land, esteemed it wit to ridicule your calling, as one well deserving, from the great numbers of its professors, contempt and derision. But they have become convinced, within the last half century, that wealth and reputation are attendant upon industry, integrity and their sister virtues, as well in the dental as in any other profession.

It is but a little more than fifty years since, James Gardette, a gentleman by birth and education, obtained a permanent residence in Philadelphia, and may justly be considered as the first who gave standing and elevation to the profession in that city. He was the first dentist whose talents and general deportment were of such a character as to make him the friend and companion of the learned and distinguished men in other professions, and the first dentist in that city whose personal virtues obtained for him a social position in the most refined and highly cultivated circles of society. I have now a letter in my possession written while I resided in London, by the honorable Joel R. Poinsett, our late minister to Mexico, recommending to me Philadelphia as the best place to settle in, mainly from the standing the profession had attained in that city, a standing given to it at that time in no other city on this continent. Let us then, in justice, acknowledge our gratitude, first, to the labors of James Gardette, and, secondly, to the accomplished dentist, scholar and gentleman, Edward Hudson, for laying in this country the foundation of professional character, upon which the most eminent, since their day, have barely been able to build superstructures corresponding to their own.

It is for you, my young friends, to preserve your calling in that respectable condition in which you find it. Although it may require less labor to do this, than to build the house into which you enter, it will, undoubtedly, be your duty to keep it in good repair. You owe this to society, which expects every man to do his duty; you owe it to those of your predecessors who have labored long and hard in the cause; you owe it to yourselves, who must stand or fall by the issue; you owe it to this institution which has a just claim to be honored by all its graduates. Such is the thorough character of a course of education here, and the competency, zeal and assiduity of your instructors, that each of you, in your practice, ought to be elevated above the ordinary ranks of your profession. Wherever you go, then, let the dignity of your deportment, your manly self-confidence, your untiring assiduity in making good your profession, your stern integrity in doing justice to all men, your pure and uncontaminated morality, preferring virtue to pleasure, your gentlemanly habits, in all respects, be your successful passport to he favor of mankind, and to the approbation of the Most High.

In order to attain to all these goods, and avoid the evils which contravene them, one particular caution deserves your regard, as constantly as your daily meals or daily devotions—it is this—avoid, peremptorily, all unworthy companions. I allude not merely to that unfortunate and much to be pitied portion of the community, whose condition and misfortune are an eternal disgrace to those who should be their protectors, but I speak here of the society of young men of doubtful sense, but of undoubted dissipation; the acquaintance and familiarity of such persons is doubly deleterious to the man of business and of honor. On the one hand, this sort of connection is always demoralizing, and, rarely ever instructive. It is one of those yawning pits into which too many young men, with thoughtless levity, eagerly plunge, engulphing valuable time, and invaluable morals. It, moreover, prevents the advantage of useful associations; it is always in the power of young men to find good company, if they will, it is particularly so with those who are known to avoid bad companions. On the other hand, evil companionship is always disreputable in the eyes of the good and the wise. They insensibly fall into the opinion of the old adage, "that birds of a feather flock together," a saying not less distinguished for its truth than its poetry. And it cannot be justly denied that time spent in such associations might be far better employed in literary reading, scientific research, healthful exercise, harmless amusement, or any of the fine arts, in the cultivation of which, during leisure hours, some of our professional brethren have shown decided talent—such as Greeenwood in painting; Hayden in mineralogy and natural history; Solyman Brown in the composition of his beautiful poem of Dentologia and in modelling for sculptor; Roper in making a large and most beautiful collection of paintings, coins, autographs and medals. But I would say let none of these pursuits, however agreeable and useful in their place, be sufficient to interfere with the faithful performance of professional duty. He who would shine in any calling, must prosecute its duties with unity of purpose. He must fix his eye upon the object of his ambition, and move towards it as steadily as the eagle pursues his track towards the sun. If he do not do this, he may chance to find himself at the evening of life, like the day-sleeping owl which flies about at twilight, in every direction, searching for scanty subsistence. Let your aims then be high and definite, if you cannot find in the personal character of any of your professional brethren, living or departed, a suitable model of imitation, put all their virtues together into one human form, into one beautiful composite order, and let that be the man of your counsel. Unless in your youthful ardor the standard of professional excellence be high, your attainments will be few and small. It is always more honorable to fall short of high aims through misfortune, than to exceed in low ones by accident. Be careful how you give credit to new methods and new inventions when they are in opposition to established principles. It is rare, indeed, that time does not prove them worse than useless, as it has done the practice of using mercurial paste for stopping teeth, which I look upon as being the greatest evil ever introduced into the profession, and I regard those who use it and those who encourage its use as being the greatest enemies to correct professional practice, and they will yet find that amalgam will fix a stain upon themselves, and upon the profession, "which neither the waters of Lethe will be able to obliterate, nor the *Letheon* to bury in oblivion," and, however dignified, learned or ingenious they may be, of this truth you may be certain, that their learning and their ingenuity has never yet reached their finger's ends—the education of which is an indispensable qualification in the good dental operator, so indispensable that no one can be good without it.

Our profession is not yet so overstocked with first rate practitioners as to have closed the gate of ambition to the honorable adventurer, there are indeed many dentists in the land, but only a few good ones : as these latter increase, the number of the former will be proportionably diminished, various causes will co-operate to produce this result, among which the graduates of this college will be conspicuous. I hope that each of you will be the means, either directly or indirectly, of inducing at least a hundred quacks to seek some more congenial employment. It will be a war of extermination, less bloody indeed, but more useful and more honorable, than that which has lately crimsoned the plains of Mexico, with the blood of thousands.

If any of you should be so fortunate, as to make a valuable discovery or improvement in professional practice, calculated to benefit the profession and its supporters, take the means of publishing it to the worthy members of your profession in such a manner that they may receive and dispense the benefit that it provides, this will give you a much higher satisfaction in after life than exhibiting in your office a gilded frame, containing a document of exclusive rights and privileges bearing the seal of the patent office at Washington, which hitherto in the greater number of instances have been obtained by designing men, arrogating to themselves that which justly belonged to others, having been by them conceived and long known and practised by intelligent members of the profession.

Have a good understanding with all, and cultivate the society of intelligent men, and particularly the experienced in your profession. I have often found conversations with such to be more profitable and valuable than reading or any other means of obtaining knowledge—but never barter professional truth for error, principle for gain, nor sacrifice the interests of your patients for the purpose of indulging in the lazy or idle habit of using that which costs the least labor, the least time, and the least money to their irreparable injury.

Be careful how you take into your hands without the concurrence or direction of a competent physician the powerful agents of late so freely and in many cases so recklessly used, and remember that you may possibly induce in some beloved and valued friend, that state which feels no change, that sleep which knows no waking. And while I would ascribe to the amiable and estimable Morton all the praise due for so great a discovery, I would in all frankness and candor say that, however valuable ether or chloroform may be in the higher and more important operations of general surgery, they are in our practice, and particularly in trivial and unimportant operations, of doubtful utility, and may yet be the means of filling many a home with sorrow and many a heart with mourning.

If you could adopt some ingenious device, by which you could be regularly reminded at the break of day, or at the rising of the sun, of the great advantage to yourselves, and others of successful practice in your calling, and at the same time, of the injuries and disadvantages of carelessness and malpractice, it would be an invaluable memento of the importance of your daily duties. In yourselves the advantage of such practice would be incalculable. They are identical with the highest object of your pursuit. If your professional labors prove successful, they will be the foundation of personal independence, the joys of domestic life, the ability of extensive usefulness as men of means, and a wide diffusion of benefactions to your fellow men. These assuredly are objects deserving of your deliberate and persevering regard. They are worthy of untiring effort, and of the purest aspirations of lofty ambition.

To others benefitted by your professional labors an honorable and faithful exercise of your art, will be an incalculable advantage also. Many thousands of human beings may be saved by your skill not merely from excruciating disease, but from the irreparable loss of the organs of mastication. To be thus eminently useful cannot fail to be a vital element of human felicity. The mere recollection of it would not fail to give exalted pleasure to declining age.

On the other hand, the disadvantages and misfortunes which must inevitably attend upon defective operations and malpractice, deserve to be known in order that they may be avoided, to the mal-operator they bring disgrace and disappointment. The disgrace he may never know except in the just and utter failure of all his hopes. This failure is nothing more nor less than professional bankruptcy, a bankruptcy of hope, a bankruptcy of reputation, a bankruptcy of anticipated fortune. From such a catastrophe, may your education, your integrity, your industry, your manly virtues, your professional dexterity, long and promptly protect you, and may the gracious smiles of superintending Providence ever irradiate your path.

I cannot better conclude these remarks, than in the beautiful and appropriate words of Dr. Charles A. Lee, in an address delivered on a similar occasion, in which he says: "We are proud of your bearing thus far, we think we see in your devotion and enthusiasm, indications of future usefulness and distinction, we believe you will play your part well as men, among men, we sympathise with you in the trials which you must necessarily encounter, we follow you in imagination forwards amidst the trying emergencies of professional life, and we glance still further on, till we see you enjoying the well earned rewards of virtue, perseverance and skill. Gentlemen, disappoint us not. Fulfil your destiny, execute your mission, carry out your purposes; with a brave heart, and manly courage, go forth in the strength of an all-wise Protector, placing your trust on high."

> "The world is all before you where to choose
> Your place of rest, and Providence your guide."

After Dr. Parmly had concluded his address, Dr. J. J. Adair, of Kentucky, rose, and in behalf of the graduates, made the following

### RESPONSE.

Fellow Graduates: Be pleased to permit me to extend to the kind father in our profession a hearty welcome, for the salutary instruction and paternal advice which he has this evening offered for our acceptance. And,

Gentlemen of the Faculty: Permit me as one, a representative, and in behalf of my fellow-graduates, to tender you our most sincere thanks, and heartfelt gratitude for the kind, assiduous and successful manner in which you have so faithfully discharged the laborious and responsible duties which have devolved upon you as professors in this institution.

We are, sensibly, aware, that should our future career demonstrate that we have fulfilled our obligations as students, with the same triumphant success with which you have discharged your duties as preceptors, the Baltimore College of Dental Surgery will never have cause to drop a tear of regret, at having begotten such sons to inherit her fame and perpetuate her memory.

But the amicable relation of pupil and preceptor, which has hitherto existed between us, you have been pleased to dissolve by the high honors which you have this evening conferred upon us; a testimonial of the estimation with which you have appreciated our attainments and proficiency; and, fellow-graduates, with this distinctive mark of favor bestowed upon us, shall we not be incited and reanimated to increased zeal and activity; to make every laudable effort, within our power, to accelerate the march of dental science; to promote and advance our profession in utility, in honorable repute, and to a more exalted position in society.

It is a matter of warm congratulation to every cultivated member of our profession, that at no former period in the history of the dental art, have the boundaries of its scientific truths been more copiously enlarged than in our present day. In Europe, Asia, Africa and America, in the islands of the sea, every where, and in all places, wherever the torch of erudition has kindled up the sparkling lights of philosophical research and inquiry, the glow and animation of medical and dental science are working out indestructible results, and building up, with imperishable materials, the temple, firm and fair, of truth and science.

But time admonishes us to be brief; therefore, in conclusion, we bid you a fond-hearted farewell, and may the comforts of earth, and the blessings of heaven accompany you through life, death, and endless eternity, is the humble invocation of your most obedient servants.

